# Circulating CRP Levels Are Associated with Epicardial and Visceral Fat Depots in Women with Metabolic Syndrome Criteria

**DOI:** 10.3390/ijms20235981

**Published:** 2019-11-27

**Authors:** Federico Carbone, Maria Stefania Lattanzio, Silvia Minetti, Anna Maria Ansaldo, Daniele Ferrara, Emilio Molina-Molina, Anna Belfiore, Edoardo Elia, Stefania Pugliese, Vincenzo Ostilio Palmieri, Fabrizio Montecucco, Piero Portincasa

**Affiliations:** 1 First Clinic of Internal Medicine, Department of Internal Medicine, University of Genoa, 6 viale Benedetto XV, 16132 Genoa, Italy; silvia.minetti@unige.it (S.M.); annamaria.ansaldo@gmail.com (A.M.A.); daniele.ferrara0292@gmail.com (D.F.); edoardo.elia93@gmail.com (E.E.); 2 IRCCS Ospedale Policlinico San Martino Genoa–Italian Cardiovascular Network, 10 Largo Benzi, 16132 Genoa, Italy; fabrizio.montecucco@unige.it; 3 Clinica Medica “A. Murri”, Department of Biomedical Sciences & Human Oncology, University of Bari Medical School, Piazza Giulio Cesare 11, 70124 Bari, Italy; emmolin.ugr@gmail.com (E.M.-M.); belfiore.murri@gmail.com (A.B.); drstefaniapugliese@libero.it (S.P.); vincenzoostilio.palmieri@uniba.it (V.O.P.); piero.portincasa@uniba.it (P.P.); 4 First Clinic of Internal Medicine, Department of Internal Medicine and Centre of Excellence for Biomedical Research (CEBR), University of Genoa, 6 viale Benedetto XV, 16132 Genoa, Italy

**Keywords:** epicardial fat thickness, visceral fat thickness, high-sensitivity c-reactive protein, leptin, gender, female

## Abstract

Sexual dimorphism accounts for significant differences in adipose tissue mass and distribution. However, how the crosstalk between visceral and ectopic fat depots occurs and which are the determinants of ectopic fat expansion and dysfunction remains unknown. Here, we focused on the impact of gender in the crosstalk between visceral and epicardial fat depots and the role of adipocytokines and high-sensitivity C-reactive protein (hs-CRP). A total of 141 outward patients (both men and women) with one or more defining criteria for metabolic syndrome (MetS) were consecutively enrolled. For all patients, demographic and clinical data were collected and ultrasound assessment of visceral adipose tissue (VFth) and epicardial fat (EFth) thickness was performed. Hs-CRP and adipocytokine levels were assessed by enzyme-linked immunosorbent assay (ELISA). Men were characterized by increased VFth and EFth (*p*-value < 0.001 and 0.014, respectively), whereas women showed higher levels of adiponectin and leptin (*p*-value < 0.001 for both). However, only in women VFth and EFth significantly correlated between them (*p* = 0.013) and also with leptin (*p* < 0.001 for both) and hs-CRP (*p* = 0.005 and *p* = 0.028, respectively). Linear regression confirmed an independent association of both leptin and hs-CRP with VFth in women, also after adjustment for age and MetS (*p* = 0.012 and 0.007, respectively). In conclusion, men and women present differences in epicardial fat deposition and systemic inflammation. An intriguing association between visceral/epicardial fat depots and chronic low-grade inflammation also emerged. In women Although a further validation in larger studies is needed, these findings suggest a critical role of sex in stratification of obese/dysmetabolic patients.

## 1. Introduction

The classical paradigm of obesity, defined by body mass index (BMI) >30 kg/m^2^, is no longer considered representative of this heterogeneous condition, characterized by many phenotypes [1]. Rather, the term “adiposopathy” has been recently coined to describe the pathological response of adipose tissue to positive caloric balance, behavioral changes and environmental factors in susceptible individuals [2]. Alongside the shift to a visceral adipose tissue (VAT) distribution and pro-inflammatory adipocytokine unbalance, the deposition of ectopic fat depots (e.g., within liver, pancreas, heart, kidney and skeletal muscle) is now considered a defining feature of adiposopathy. The growing evidence of individual variation in body fat distribution also raised the interest toward the susceptibility of visceral fat storing to genetic factors [3], including racial and sex differences [4,5]. Especially, sexual dimorphism accounts for significant differences in visceral fat mass and distribution, women being characterized by greater BMI with prevalent subcutaneous distribution. This has important clinical implications as visceral and subcutaneous fat greatly differ in terms of function and response to weight gain. Sex differences were also reported in the chronic low-grade inflammatory response underlying obesity. Full adipose tissue profiling in both experimental and clinical studies demonstrated sex difference in insulin resistance and inflammatory response. More specifically, women seem to be protected from macrophage infiltration into the adipose tissue [6], and show a stronger association between adiposity and C-reactive protein (CRP) [7].

Conversely less is known about the role of epicardial fat tissue thickness (EFth) and function. EFth correlates with metabolic syndrome and its relative clinical features, but the strength of this association is less than half of that with visceral adipose tissue [8]. Sex-related differences in the crosstalk between visceral, ectopic fat and circulating inflammatory molecules are also poorly investigated [9]. Nevertheless, this is a very critical point because epicardial fat is increasingly described as a metabolic active organ, with detrimental effects on surrounding tissues (i.e., coronary arteries and myocardium) [10]. There is indeed an urgent need to understand, which are the determinants of epicardial fat expansion and dysfunction, whether sex-related differences in epicardial fat deposition exist and how the crosstalk between visceral and ectopic fat depots occurs. With this aim, a cohort of outward patients with one or more defining criteria for MetS have been analyzed here. We particularly focused on the potential impact of sex in visceral and epicardial fat deposition, also considering if any correlation with adipocytokines and high-sensitivity CRP (hs-CRP) exists.

## 2. Results

### 2.1. Men and Women Have a Similar Metabolic Profile

The characteristics of the overall cohort are shown in Table 1.

Median age of patients was 56 years with almost equal distribution across sex (men 46.4%). Concerning metabolic syndrome criteria, hypertension was highly frequent (80.0%), whereas the prevalence of glucose intolerance was lower (impaired fasting glucose 0.8% and diabetes 13.6%). Overall, more than half of patients had 1 or 2 MetS defining criteria (32.7% and 39.2%, respectively), whereas MetS was recorded in 28.0% of patients. Once categorized for sex, men showed a weak increase of blood pressure values, waist circumference/weight measurement and glycolipid profile, but not BMI (Table 2).

**Figure 1:**
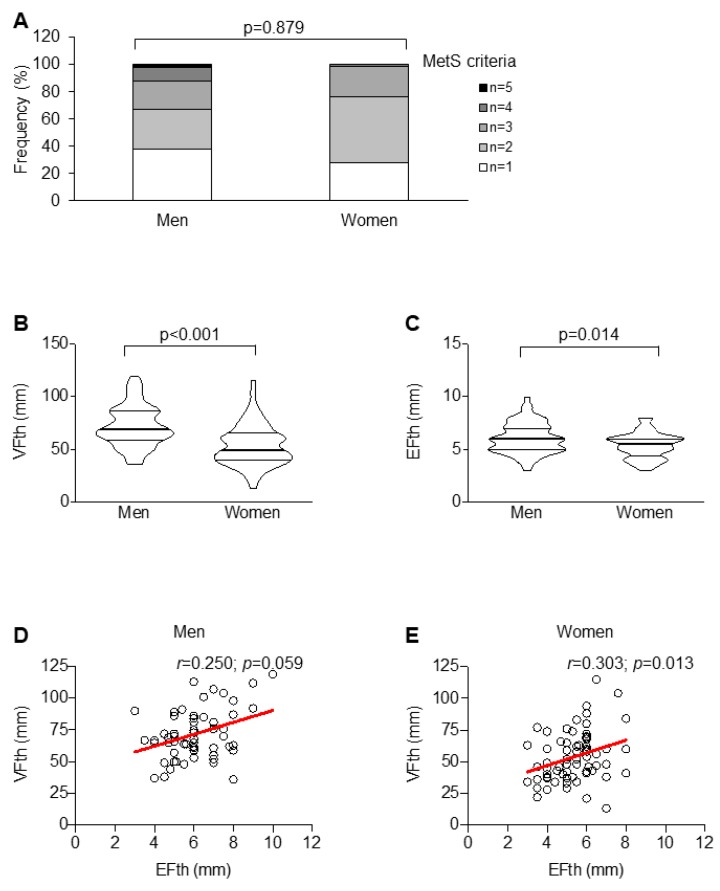
Metabolic differences and adipose tissue distribution across sex. Sex distribution in term of numbers of metabolic syndrome (MetS) criteria (**A**). Sex-related differences in the extent of visceral fat thickness (VFth) (**B**) and epicardial fat thickness (EFth) (**C**) and their correlation in men (**D**) and women (**E**).

Of interest, the prevalence of MetS did not differ across sex (Figure 1A). 

### 2.2. Men Have Increased Ectopic Fat Depots but Reduced Circulating Adipocytokine Levels as Compared to Women

In our cohort of patients with at least one MetS criterion, men were characterized by greater VFth and EFth (*p*-value < 0.001 and 0.014, respectively) as compared to women (Figure 1B,C). However, only in women, VFth and EFth significantly correlated with each other (*r* = 0.303; *p* = 0.013) (Figure 1D,E).

**Figure 2:**
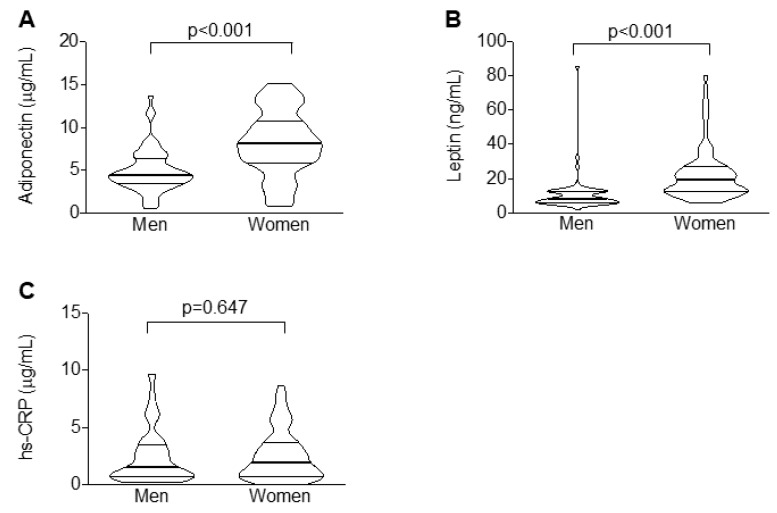
Women are characterized by greater serum levels of adipocytokines but not hs-CRP. Violin plots illustrating the median values across sex of adiponectin (**A**), leptin (**B**), and high-sensitivity C-reactive protein (hs-CRP) (**C**).

Conversely, women showed increased levels of adiponectin and leptin (*p*-value < 0.001 for both; Figure 2A,B), whereas the concentrations of hs-CRP were similar across sex (Figure 2C).

### 2.3. Only in Women, Circulating Levels of Leptin and CRP are Associated with Visceral and Ectopic Fat Depots

**Figure 3:**
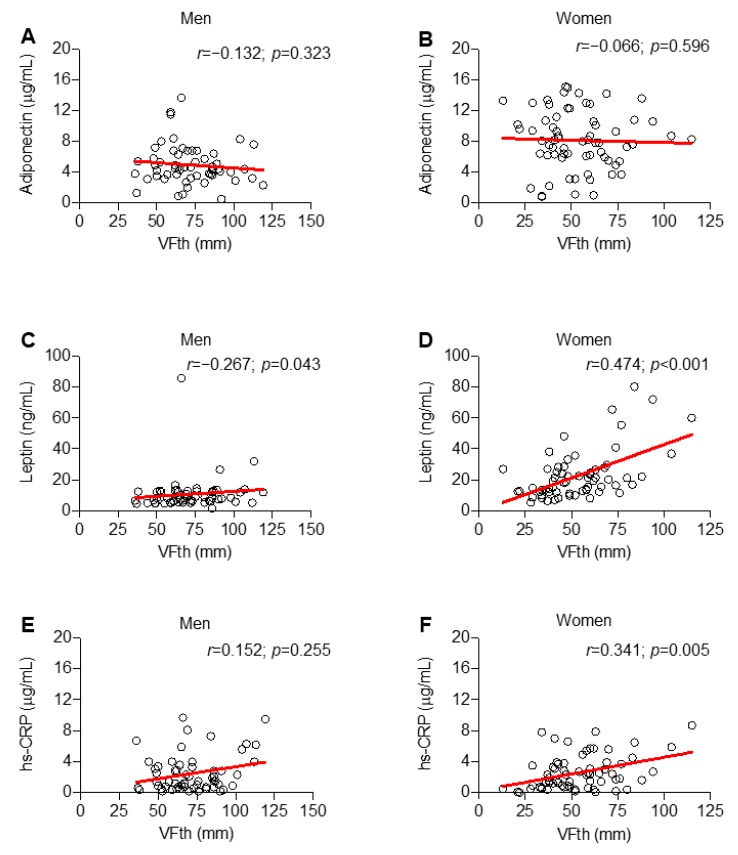
Only in women, hs-CRP correlates with the extent of visceral fat thickness (VFth). Scatter plot illustrating the correlation of VFth with serum biomarkers across sex: adiponectin (**A**,**B**), leptin (**C**,**D**) and hs-CRP (**E**,**F**).

Considering potential association between circulating levels of inflammatory molecules and fat distribution in men and women, no correlation was found between adiponectin and VFth (Figure 3A,B).

**Figure 4:**
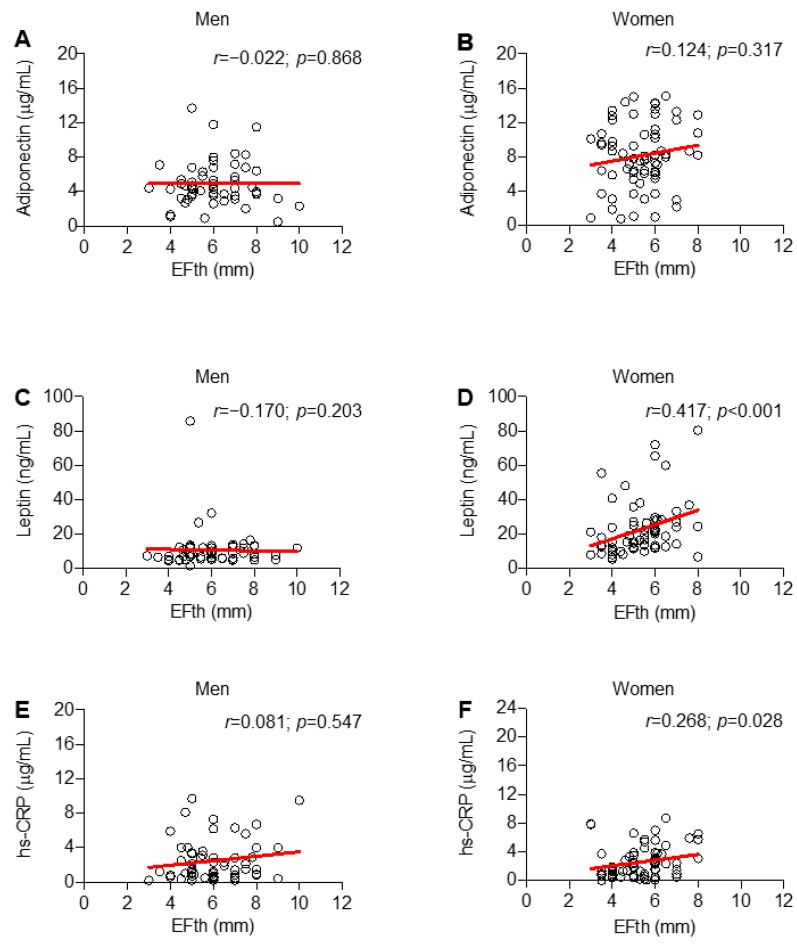
Only in women, leptin and high-sensitivity C-reactive protein (hs-CRP) correlate with the extent of epicardial fat thickness (EFth). Scatter plot illustrating the correlation of EFth with serum biomarkers across sex: adiponectin (**A**,**B**), leptin (**C**,**D**) and hs-CRP (**E**,**F**).

Conversely, the adipocytokine leptin correlated with VFth both in men and women (*p* = 0.043 and < 0.001, respectively) (Figure 3C,D). Hs-CRP correlated with VFth only in women (*r* = 0.341; *p* = 0.005) (Figure 3E,F). Considering EFth, no correlation between serum adiponectin and this variable was observed in both men and women (Figure 4A,B).

Only in women, significant correlations between EFth and leptin (*r* = 0.417; *p* < 0.001) and hs-CRP (*r* = 0.268; *p* = 0.028) were demonstrated (Figure 4C–F).

As difference in systemic biomarkers occurred only in women, linear regression analyses to test the independent associations were performed only in this sub-group. Both serum leptin and hs-CRP were independently associated with VFth also after adjustment for age and MetS criteria (Table 3).

Conversely, hs-CRP was independently associated with EFth in univariate analysis (B 0.12 [95%CI 0.02–0.25]; *p* = 0.026) but not in the adjusted analysis (Table 3).

## 3. Discussion

The present study further supports current evidence about the role of sex in determining adipose tissue distribution and inflammatory status. Although the extent of both visceral and epicardial fat depots was greater in men, only in women a correlation between serum inflammatory molecules and fat depots was observed. This may be not surprising as sexual dimorphism is already known to influence adipose distribution, systemic inflammation, cardio-metabolic disorders and clinical response to bariatric surgery as well [7,11,12]. Such differences in adipose tissue distribution may be ascribed to a different lipoprotein lipase activity and estrogen receptor expression [5]. Estrogens also modulate food intake and energy expenditure, potentially inducing anti-obese effects. Due to the high aromatase content, especially subcutaneous adipose tissue (SAT) contributes to this cardio-metabolic protection, which is lost in post-menopausal women [13]. Concerning sex-related differences in ectopic fat depots, the higher EFth observed in men may be ascribed to the well-known association existing between VAT extent and ectopic fat deposition. Sexual dimorphism in epicardial fat occurs in aging rats and this may be ascribed to differences in adipocytokines and obesity-related genes [14,15]. Also in human beings, the SWAN Cardiovascular Fat Ancillary Study reported increased EFth and coronary artery calcification in post-menopausal women, in association with decreased estradiol levels [16,17].

Viewed from an allostatic perspective, ectopic fat deposition occurs when storage capacity is exceeded over a defined limit. A genetic control of this limit would exist at both population and individual levels, with a significant role of age and sex [18,19,20]. However, VAT extent by itself is unlikely the only determinant of ectopic fat distribution and then is not sufficient to explain the new concepts of “obesity paradox” and “adiposopathy” [1,21]. In line with other studies, our results rather suggest a sex-specific correlation of chronic low-grade systemic inflammation with visceral and ectopic fat depots. Although preliminary, this finding may be considered as a strength of this study and could be a flywheel for future researches. More specifically, hs-CRP showed the strongest association with adipose depots. Overall, those data further claim for a role of hs-CRP in cardio-metabolic risk stratification, whereas it remains largely unexplained whether and how adipocytokines contribute to adipose tissue extent and function. Many researches on different models have attempted to address this point. A stronger association between obesity and inflammation has been already observed in women than men, especially for hs-CRP. A recent meta-analysis enrolling more than eighty thousand obese patients from 51 cross-sectional studies confirmed a correlation between BMI and serum hs-CRP, which was stronger in Caucasian women [22]. High inflammation in women was later reported in the HERMEX study [23]. Our findings are in line with this sex-specific link between visceral and epicardial fat through chronic low-grade inflammatory processes. We might speculate on pathophysiological mechanisms underlying such associations. Estrogens are widely associated with anti-inflammatory effects [24], so that insulin resistance and ectopic fat deposition are more frequently observed in post- than pre-menopausal women [25]. Nevertheless, even in post-menopausal women, hormone replacement therapy is associated with higher levels of hs-CRP [26,27]. Similarly, sexual dimorphism also modulates adipocytokine metabolism [28,29]. In line with our results, circulating levels of both leptin and adiponectin increased in women as compared to men [30,31]. Biological reasons for those differences are still unknown, but they seem human-specific as in rodents they are less apparent or even opposite [32]. 

This study has several limitations. Firstly, due the relatively small size of our cohort, the study may not be representative of the general population and thereby does not take into account all potential variables influencing EFth. In addition, the cross-sectional design of the study does not only allow to provide any relationship with long-term outcomes. Secondly, we have to acknowledge that the reproductive status of patients was not presented in this study, as we did not collect information about physiological/pharmacological history and hormonal background. Thirdly, computerized tomography and magnetic resonance would be the gold standard method to quantify adipose tissue depots. However, ultrasound is increasingly being validated and not burdened by high cost and limitation due to accessibility and radiation exposure. In our context, ultrasonography offers a wide range of opportunities in clinical setting, by providing accurate, non-invasive, and reproducible measurement of adipose tissue depots in different districts.

Finally, here we focused only of EFth, without considering other ectopic fat depots, especially hepatic steatosis, for which a sex-related differences have been widely reported. However, an extensive evaluation of whole-body fat depots—which should also include intramuscular, pancreatic and renal ones among others—is out of scope for the present study. Too many variables should have been considered, due to the differences between ectopic fat tissues in terms of vascularization, metabolic function and tissue environment. Future larger studies can discriminate between the unicity of each tissue and the common underlying pathways leading to ectopic fat deposition, including the sex-related ones.

## 4. Material and Methods

### 4.1. Patients

In this cross-sectional observational monocentric study, we enrolled 141 consecutive patients aged 30 to 65 years referred for one or more diagnostic criteria for MetS to the Clinica Medica “A. Murri”, Department of Biomedical Sciences and Human Oncology, University of Bari Medical School (Bari, Italy). Exclusion criteria were systolic blood pressure (sBP) > 200 mm Hg or diastolic blood pressure (dBP) ≥ 120 mm Hg; reduction of left ventricular ejection fraction; cardiovascular disease (myocardial infarction or stroke within 6 months, angina, coronary revascularization, systolic heart failure); atrial fibrillation or flutter as well as other dysrhythmias; inflammatory bowel disease; severe psychiatric disorders; chronic kidney disease (glomerular filtration rate < 60 mL/min); secondary hypertension; peripheral arterial disease; alcoholism or use of illicit drugs; severe hepatic diseases; history of cancer; use of immunosuppressive drugs, chemotherapy, or radiotherapy; or inability to understand or adhere to study procedures.

In a second step we have further excluded patients with serum levels of hs-CRP above 10 µg/dL, in order to avoid potential bias due to other concomitant inflammatory conditions. The Ethics Committee of University Hospital Policlinico in Bari approved this protocol, performed in accordance to the guidelines of the Declaration of Helsinki. Patients gave informed consent before entering in the study (study number 5408, protocol number 0013869; approved by AOUCPG23/COMET/P on 7 July 2017).

### 4.2. Study Endpoints and Statistical Power Calculation

The primary outcome was to determine sex-related differences in EFth. According with the power study calculation based also on previous studies [33] our sample size (*n* = 125) was able to detect differences in EFth across sex with a power of 80% and a two-sided alpha error of 5%. Secondary endpoints include the potential impact of sex in the inflammatory crosstalk between visceral and epicardial adipose tissues (i.e., serum levels of adipocytokines and hs-CRP).

### 4.3. Data Collection and Assessment

For all patients, clinical data were collected, including, comorbidities and anthropometric measures. Waist circumference was measured at the midpoint between the lower border of the rib cage and the iliac crest. Visceral obesity was then diagnosed in the presence of waist circumference ≥ 96 cm in men and ≥ 80 cm in women, as recommended for the Mediterranean/European population [34]. The diagnosis of arterial hypertension was based on sBP ≥ 140 mmHg and/or dBP ≥ 90 mmHg (measured three times within 30 min, in the sitting position and using a brachial sphygmomanometer), or use of blood-pressure-lowering agents. The diagnosis of impaired fasting glucose and of type 2 diabetes (T2DM) was based on the revised criteria of the American Diabetes Association, using a value of fasting blood glucose ≥ 100 to < 126 mg/dL, and ≥126 mg/dL or the use of insulin or oral hypoglycemic agents, respectively [35]. A 12-h overnight fasting blood sample was drawn to determine hematological and biochemical profile. As surrogate marker of visceral adipose tissue dysfunction, we calculated the visceral adiposity index (VAI), through a validated mathematical model that uses both anthropometric (body mass index [BMI] and waist circumference) and functional (triglycerides [TAG] and high-density lipoprotein cholesterol [HDL-c]) simple parameters [36,37]. Finally, diagnosis of MetS was made based on the harmonized MetS criteria [34].

### 4.4. Ultrasound Assessment of Fat Depots

Visceral fat was measured with the Hitachi Noblus-E ecocolordoppler (Hitachi Medical, Tokyo, Japan) and Logiq E9 (GE, Healthcare, Chicago, IL, USA) ultrasound equipment equipped with a 3.5 MHz convex probe.

Epicardial fat thickness was measured with the M5S 7.5 MHz convex probe while the ML6-15 linear probe was used for the measurement of carotid IMT and plaques, Logiq E9 (GE, Healthcare,). All ultrasound examinations and diagnoses were performed by a trained internal medicine specialist. The visceral fat thickness was measured from the center of the left hepatic lobe [38,39].

### 4.5. Statistical Analysis

Analyses were performed with IBM SPSS Statistics for Windows, Version 23.0 (IBM CO. Armonk, NY, USA). Categorical data are presented as absolute (and relative) frequencies and analyzed by Chi square or Fisher′s exact test, as appropriate. Continuous variables have been expressed as median and interquartile range (IQR) as the normality assumption was not demonstrated. Intergroup comparisons were then drawn by Mann–Whitney test, whereas correlations were investigated through Spearman’s rank correlation coefficient. Adjusted linear and logistic regressions have been used to evaluate the independency of the association between inflammatory biomarkers and adipose tissue depots. For all statistical analyses, a 2-sided *p*-value < 0.05 has been considered as statistically significant.

## 5. Conclusions

In conclusion, our study supports a close association between visceral and epicardial fat depots in the subset of women with at least one MetS criterion. This crosstalk would be mediated by a chronic low-grade inflammatory status. Since ectopic lipid accumulation precedes the metabolic and atherosclerotic complications, identifying fat-related inflammatory biomarkers might have a clinical utility in stratifying obese/dysmetabolic patients and eventually in CV risk estimation. This approach might then optimize intervention outcomes and produce a more personalized approach [40].

## Figures and Tables

**Table 1:** Baseline characteristics in the overall cohort.

Parameters	Overall Cohort (*n* = 125)
**Clinical**	
Age, yr. [IQR]	56 (49–62)
Men, no. (%)	58 (46.4)
Active smokers, no. (%)	21 (16.8)
Hypertension, no (%)	100 (80.0)
IFG, no (%)	1 (0.8)
T2DM, no (%)	17 (13.6)
sBP, mmHg [IQR]	130 (120–135)
dBP, mmHg [IQR]	80 (72–85)
Waist circumference, cm [IQR]	99 (93–108)
Weight, Kg [IQR]	77 (68–90)
BMI, Kg/m^2^ [IQR]	27.2 (25.1–30.3)
MetS criteria	
1	41 (32.7)
2	49 (39.2)
3	27 (21.6)
4	7 (5.6)
5	1 (0.8)
MetS	35 (28.0)
**Ultrasound assessment**	
EFth, mm [IQR]	5.6 (4.8–6.5)
VFth, mm [IQR]	61 (46–76)
Hepatic steatosis, no. (%)	85 (68.0)
**Biochemistry**	
Serum total-c, mg/dL [IQR]	194 (168–219)
Serum LDL-c, mg/dL [IQR]	111 (83–129)
Serum HDL-c, mg/dL [IQR]	60 (50–71)
Serum TAG, mg/dL [IQR]	101 (71–139)
Fasting glycaemia, mg/dL [IQR]	89 (83–99)
VAI, *n* [IQR]	1.3 (0.8–1.9)

sBP: systolic blood pressure; dBP: diastolic blood pressure; BMI: body mass index; MetS: metabolic syndrome; EFth: epicardial fat thickness; VFth: visceral fat thickness; LDL-c: low-density lipoprotein cholesterol; HDL-c: high-density lipoprotein cholesterol; TAG: triglycerides; VAI: visceral adiposity index.

**Table 2:** Baseline clinical/biochemical characteristics across sex.

Clinical Data	Men (*n* = 66)	Women (*n* = 75)	*p*-Value
Age, yr. (IQR)	56 (49–60)	57 (47–63)	0.335
Active smokers, no. (%)	11 (19.0)	10 (14.9)	0.634
sBP, mmHg (IQR)	130 (125–135)	125 (115–136)	0.017
dBP, mmHg (IQR)	80 (80–89)	80 (70–85)	0.010
Waist circumference, cm (IQR)	103 (95–110)	97 (90–102)	0.005
Weight, Kg (IQR)	86 (76–93)	70 (63–80)	< 0.001
BMI, kg/m^2^ (IQR)	28.4 (25.7–30.4)	26.7 (24.8–30.0)	0.216
Biochemistry			
Serum total-c, mg/dL (IQR)	186 (161–210)	197 (170–225)	0.042
Serum LDL-c, mg/dL (IQR)	110 (81–127)	116 (89–133)	0.259
Serum HDL-c, mg/dL (IQR)	53 (43–59)	69 (60–77)	< 0.001
Serum TAG, mg/dL (IQR)	118 (85–169)	90 (65–126)	0.001
Fasting glycaemia, mg/dL (IQR)	92 (86–104)	87 (82–98)	0.013
VAI, n (IQR)	1.4 (0.9–2.2)	1.3 (0.8–1.7)	0.130

sBP: systolic blood pressure; dBP: diastolic blood pressure; BMI: body mass index; total-c: total cholesterol; LDL-c: low-density lipoprotein cholesterol; HDL-c: high-density lipoprotein cholesterol; TAG: triglycerides; VAI: visceral adiposity index.

**Table 3:** Linear regression showing the association of adipose tissue depots with inflammatory biomarkers in women.

Variables	Univariate		Adjusted	
VFth	B (95% CI)	*p*-Value	B (95% CI)	*p*-Value
Age	0.69 (0.22–1.15)	0.004	0.33 (–0.09–0.76)	0.122
MetS criteria	13.37 (7.84–18.91)	< 0.001	9.46 (3.78–15)	0.001
Leptin	0.15 (0.02–0.28)	0.025	0.14 (0.03–0.24)	0.012
hs-CRP	3.52 (1.47–5.57)	0.001	2.61 (0.75–4.47)	0.007
**EFth**	B (95% CI)	*p*–value	B (95% CI)	*p*-value
Age	0.05 (0.02–0.08)	< 0.001	0.03 (0.01–0.06)	0.006
MetS criteria	0.85 (0.51–1.18)	< 0.001	0.61 (0.30–0.12)	0.001
Leptin	0.01 (–0.00–0.01)	0.116	-	-
hs-CRP	0.12 (0.02–0.25)	0.026	0.05 (–0.06–0.17)	0.390

Vth: visceral fat thickness; MetS: metabolic syndrome; EFth: epicardial fat thickness; hs-CRP: high-sensitivity C-reactive protein.
